# Genome-wide identification of the *GLK* gene family in wheat (*Triticum aestivum* L.) and analysis of expression responses in different environments

**DOI:** 10.1186/s12864-025-12456-2

**Published:** 2026-01-20

**Authors:** Huaqing Li, Teng Li, Yang Liu, Yang Yu, Yesong Tian, Pengxia Guo, Yan zheng, Donghong Min, Xiaohong Zhang

**Affiliations:** College of Agronomy, State Key Laboratory of Crop Stress Biology for Arid Areas, Northwest A&F University, Yangling, Shaanxi 712100 China

**Keywords:** Wheat, *Golden2-like* (*GLK*), Family analysis, Photosynthesis

## Abstract

Wheat plays a crucial role in ensuring global food security, and the *GLK* family of transcription factors is an important regulator of chloroplast development, chlorophyll synthesis, photosynthesis, and stress tolerance. However, there are few genome-wide studies of the *GLK* genes in wheat, especially regarding their roles in photosynthesis and phosphorus (Pi) deficiency. In this study, 173 *GLK* transcription factors (TFs) were identified in wheat and combined with bioinformatics analysis and statistical methods. The wheat *GLK* family was divided into eight subfamilies (I-VIII) based on phylogenetic relationships and motif composition. The *TaGLK* genes had mainly undergone segmental duplication during their evolution and had been subjected to strong selective pressure for purification. Both subcellular localization predictions and experimental results suggest that the wheat GLK protein was primarily localized in the cell nucleus. The *TaGLK* genes showed a higher degree of collinearity with those of maize in the gramineous family, suggesting that the two were more closely related in terms of their evolutionary relationship. Transcriptome data analysis results indicated that the expression patterns of wheat *GLK* genes were significantly different in various tissues, and there were significant differences in the amount of expression. Meanwhile, RT–qPCR analysis revealed that *TaGLKs* genes expression were induced by light, dark, Pi starvation, and drought stress. Among them, members of subfamily VI were structurally conserved and may be involved in the development of chloroplasts and may positively regulate photosynthesis and chlorophyll biosynthesis, whereas members of subfamily VIII had a conserved motif (LHEQLE) and may be involved in the stress response to phosphorus deficiency. These findings provide valuable information for further studies on the function of *TaGLK* genes and their potential roles in light signal sensing, chloroplast development, and abiotic stress response.

**Supplementary Information:**

The online version contains supplementary material available at https://doi.org/10.1186/s12864-025-12456-2.

## Introduction

Ensuring food security was dependent on improving wheat''s photosynthetic efficiency, especially considering population growth and the decline of arable land. However, this strategy was being hindered by climate warming and increasingly severe droughts [1–4]. Additionally, the excessive application of Pi fertilizers results in crops absorbing only 20–30% of them. Furthermore, Pi loss exacerbates environmental pollution [5, 6].

The *GLK* transcription factor was a key regulatory node within the GARP superfamily of MYB transcription factors. In terms of its structure, the C-terminal region contained both a Myb DNA-binding domain (DBD) and a unique GCT box domain, which were both essential for homodimerization or heterodimerization [7–9]. It was initially identified in C4 plants such as maize and was shown to stimulate chloroplast development in bundle sheath and mesophyll cells. This conserved role was later confirmed in C3 species, often with functional redundancy [10, 11]. The *GLK* gene has been identified in maize (*Zea mays*) [12], rice (*Oryza sativa*) [13], and *Arabidopsis thaliana* (*A thaliana*) [14], with most studies focusing on abiotic stresses such as cold and drought. The *GLK* gene functions independently and cooperates with other genes [15, 16]. The *glk1glk2* double mutant exhibited a pale green color, impaired chloroplast development, reduced thylakoid stacking. Overexpression of *GLK* genes increased the accumulation of genes related to photosystem proteins (LHCB1.2 and LHCA1) and chlorophyll (Chla and Chlb) biosynthesis, as well as regulating nuclear gene expression [17, 18]. Although the functional roles of *GLK* genes in chloroplast development, chlorophyll biosynthesis, photosynthetic efficiency had been extensively characterized in various crop species, their specific functions and regulatory mechanisms in wheat remained unclear.

In addition to its involvement in chloroplast development, *GLK* played a significant role in responses to abiotic stress. The *GLK* gene positively regulated drought tolerance. overexpressing plants sustain less damage during periods of drought compared to wild-type plants, whereas gene silencing results in more severe injury [19–22]. The *GLK* gene was also involved in the response to Pi deficiency. Under low-Pi conditions, root photosynthesis was promoted by *GLK*, significant accumulation of GLK proteins was induced, and their stability was enhanced [23, 24]. It was well known that *GLK* genes regulate abiotic stress responses in different species [25, 26]. However, their specific functions and regulatory mechanisms in wheat were poorly understood.

Through genome-wide analysis, we identified 173 members of the *TaGLK* family in wheat and characterized their gene structures, evolutionary relationships, and promoter *cis*-elements. Transcriptome data revealed that *TaGLK* genes exhibited distinct, tissue-specific expression patterns. Furthermore, RT-qPCR analysis demonstrated that certain *TaGLK* genes were responsive to light, dark, drought, and low-Pi stress, suggesting their potential involvement in indirectly affecting the efficiency of photosynthetic light and dark reactions, as well as in responses to drought and low-Pi stress. This study also provided the first prediction of interacting proteins and microRNAs (miRNAs) for the *GLK* family in wheat. Therefore, this study aimed to screen key candidate genes involved in chloroplast development and stress response by conducting a genome-wide identification, phylogenetic analysis, and expression profiling of the wheat *TaGLK* gene family under abiotic stress, thereby laying a foundation for further in-depth exploration of its molecular regulatory network.

## Materials and methods

### Material and treatment

To investigate *TaGLK* expression under various conditions, we analyzed the expression patterns of four *TaGLK* genes in response to specific treatments. Initially, seeds of the Chinese spring wheat variety were soaked in 2% (v/v) hydrogen peroxide (H₂O₂) for 18 h. Subsequently, they were rinsed with distilled water and placed on moist filter paper. To ensure consistent germination, the seeds underwent 1 day of dark germination, followed by 1 week in a light incubator at 18–22 °C with a 16 h/8 h (light/dark) cycle. 1-week-old seedlings were subjected to three distinct treatments. For the light/dark treatment, seedlings were exposed to either 12,000 lx or 0 lx, with samples collected at 0, 6, 12, 24, and 48 h. For drought stress, seedlings were treated with a 20% (w/v) polyethylene glycol 6000 (PEG 6000) solution. For Pi stress, seedlings were subjected to low (LP: 10 µmol/L), normal (NP: 200 µmol/L), and high (HP: 625 µmol/L or 1 mmol/L) Pi conditions. For the drought and phosphate stress treatments, samples were collected at 0, 1.5, 3, 6, 12, 24, and 48 h post-treatment. All samples were immediately frozen and stored at −80 °C for subsequent RNA extraction. The expression levels of the target *TaGLK* genes were examined using quantitative RT-qPCR, with the expression of a wheat actin gene used for normalization [27]. All samples were subjected to three biological replicates.

### Identification and characterization of *TaGLK*

To identify members of the wheat *GLK* transcription factor family, both the *Triticum aestivum* (*T aestivum*) genome sequence (FASTA format) and its corresponding genomic annotations (GFF3 format) were retrieved from the Ensembl Plants database (https://plants.ensembl.org/info/data/ftp/index.html) [28]. At the same time, the Hidden Markov Model (HMM) data for the GLK domain was obtained from the InterPro database v105.0 (https://www.ebi.ac.uk/interpro/) [29]. To extract the CDS sequences from the genome, the ''Gff3 Sequence Extract'' function in TBtools-II v2.356 was used, and these were then translated into protein sequences using the ''Batch Translate CDS'' function [30]. The candidate TaGLKs were identified using a dual search strategy. Firstly, a BlastP search was performed using the known *A. thaliana* and maize GLK1 and GLK2 protein sequences as queries, with an E-value cut-off of ≤ 1e-5 [7, 17, 30]. Secondly, a separate HMM search was conducted in TBtools-II using the GLK HMM profile with an E-value cut-off of < 0.02. The results from both methods were integrated, and, for genes with multiple transcripts, only the longest isoform was retained. Lastly, an analysis of the SMART (https://smart.embl.de/) and NCBI Conserved Domain Database (CDD) tools (https://www.ncbi.nlm.nih.gov/cdd/) confirmed their identity as bona fide TaGLK proteins [31, 32].

### Basic information on *TaGLKs* and phylogenetic analysis with other species

To gain further insight into the TaGLK family, the physicochemical properties, subcellular localization, and phylogenetic relationships of TaGLK proteins were analyzed. The hydrophobicity, relative molecular mass, and isoelectric point of the TaGLK proteins were predicted using the ExPASy website (https://web.expasy.org/protparam/) [33]. Subcellular localization was determined using the BUSCA Website (https://busca.biocomp.unibo.it/) [34]. Multiple sequence alignment of the GLK proteins in wheat, maize, and *A thaliana* were performed using the ClustalW method in MEGA11 software. An evolutionary tree was constructed using the Neighbor-Joining (NJ) method with 1000 bootstrap replicates. Finally, branch colors, groups, and species classifications were added to the evolutionary tree using the Evolview v2 website (https://evolgenius.info/evolview-v2/#mytrees/) [35].

### Chromosome mapping

To determine the chromosomal location of the *GLK* gene in wheat, the chromosomal mapping was conducted. The chromosomal locations of the *TaGLK* genes were determined by extracting positional data from the Ensembl Plants database and calculating chromosome lengths using the Fasta Statistics function in TBtools-II. The results were then visualized using the MG2C v2.1 online tool (http://mg2c.iask.in/mg2c_v2.1/) [36].

### Phylogenetic relationships, gene structure, and conserved motif analysis

To characterize the *TaGLK* gene family, we analyzed their phylogenetic relationships, gene structures, and conserved motifs. Primarily, we performed a multiple sequence alignment of the TaGLK proteins and constructed a phylogenetic tree using the Neighbor-Joining (NJ) method in MEGA11 software with 1000 bootstrap replicates. Additionally, we determined the exon–intron structures, including the 5'' and 3'' untranslated regions (UTRs), using data from the Ensembl Plants database, visualizing them with Evolview v2 software [35]. Finally, we identified the conserved motifs using the online MEME v5.0.5 suite (https://meme-suite.org/meme/), setting the maximum number of motifs to 20 [37].

### Collinearity and protein interaction network analysis

To clarify the evolutionary origin and functional potential of the *TaGLK* family, a series of collinearity analyses was conducted. Collinearity within the wheat genome was assessed by performing a BLASTP v2.14.0 search (E-value ≤ 1 × 10⁻^5^) to identify paralogous gene pairs. Segmental duplication events were then identified from these results using MCScanX. The collinear relationships were then mapped and visualized using Circos v0.69–9. The selective pressure exerted on these duplicated genes was evaluated by calculating their non-synonymous to synonymous substitution (Ka/Ks) ratios using the relevant tool in TBtools-II [30].

To analyze interspecies synteny, the wheat genome was compared to those of maize and *A thaliana* using the One Step MCScanX-Super Fast plugin in TBtools-II. The synteny files generated from these comparisons were consolidated using the ''Text Merge for MCScanX'' function and ultimately visualized using the ''Multiple Synteny Plot'' tool. Furthermore, to elucidate the potential functional roles of the TaGLK proteins, a protein–protein interaction network was predicted. This was achieved by using orthologs from the well-characterized model plant *A thaliana* in the STRING v12.0 database and applying a minimum interaction confidence score of 70% [38]. The network data retrieved from STRING were rendered using Cytoscape v3.10.0 software [39].

### *Cis*-acting element analysis and miRNA prediction

To understand the potential expression patterns, stress response pathways, and co-regulatory networks of the *TaGLK*, their promoters were analyzed. The promoter sequences (extracted from 2,000 bp upstream of the ATG start codon) were submitted to the PlantCARE website (http://bioinformatics.psb.ugent.be/webtools/plantcare/html/) for *cis*-element prediction [40], and the results were visualised using TBtools-II. Potential miRNAs in *TaGLK* genes were predicted using the psRNATarget server(https://www.zhaolab.org/psRNATarget/) [41]. The psRNATarget server was used to predict post-transcriptional regulation by identifying miRNAs targeting the *TaGLK* genes, and these regulatory relationships were visualized as a network in Cytoscape v3.10.0 [39, 41]. The GSDS v2.0 website (https://gsds.gao-lab.org/index.php) was used to draw the different splice variants of the genes [42].

### Gene family expression patterns and real-time quantitative PCR (RT-qPCR) analysis

To investigate the expression patterns of *TaGLK* genes, we analyzed publicly available RNA-seq data from multiple tissue types downloaded from the Wheat Omics website (http://wheatomics.sdau.edu.cn/) [43]. We generated a heatmap of gene expression levels based on transcripts per million (TPM) values using TBtools-II software. Further examination of *TaGLK* expression under specific stress conditions was conducted using RT-qPCR. Four *TaGLK* family members were examined in seedlings exposed to light, darkness, drought, and varying Pi concentrations. Total RNA was extracted from these samples using the TRIzol method. The quality of the RNA was verified by assessing the concentration and purity using a NanoDrop One Spectrophotometer, and the integrity was confirmed via 1.0% agarose gel electrophoresis. Only high-quality RNA samples (with an OD260/OD280 ratio of 1.8–2.0) were reverse transcribed into cDNA using a PrimeScript™ RT kit with gDNA Eraser (TaKaRa, China) according to the manufacturer''s instructions. The resulting cDNA was stored at −20 °C. The RT-qPCR experiments were performed using gene-specific primers (Table S11). The relative expression levels of the *TaGLK* target genes were calculated using the 2^(-ΔΔCt) method. The data were organized in Microsoft Excel 2024, and statistical significance was determined using one-way ANOVA followed by Tukey’s multiple comparison tests. All data are presented as mean ± SD, a significance threshold of p < 0.05 was used, with different lowercase letters indicating significant differences. All experimental groups included three biological replicates.

### Subcellular localization analysis of TaGLK40 and TaGLK144 proteins

To investigate the subcellular localization of TaGLK40 and TaGLK144, transient expression assay in *Nicotiana tabacum* were conducted in this study. The proteins were expressed as C-terminal fusions to green fluorescent protein (GFP) under the control of the cauliflower mosaic virus 35S promoter (CaMV 35S promoter) in the pCAMBIA1302-GFP vector. These recombinant plasmids, alongside an empty vector control, were introduced into the GV3101 strain of *Agrobacterium tumefaciens* and subsequently infiltrated into the leaves of 4-week-old tobacco plants. Subcellular localization was assessed by visualizing GFP fluorescence with a Leica laser confocal microscope.

## Results

### Identification and basic information of the *TaGLK* gene family

This investigation used the HMM to analyze the *T. aestivum* genome and identify candidate GLK proteins with the distinctive MYB DNA-binding domain. These initial candidates were then refined by selecting the longest transcript for each gene and by validating the presence of the conserved domain through homologous sequence comparison with known GLKs from other species. Analyses were also performed using SMART and NCBI-CDD. Ultimately, this process identified 173 *TaGLK* genes, which were systematically named *TaGLK1* to *TaGLK173*. Table S1 details the basic parameters of these proteins, including amino acid length, molecular weight, and isoelectric point. According to subcellular localization predictions, the majority of TaGLK proteins (164/173) were localized to the nucleus, which was consistent with their role as transcription factors. A smaller number were predicted to be chloroplast (6/173), mitochondrion (2/173), or associated with the mitochondrial membrane (1/173). Multiple sequence alignment of the protein sequences revealed that wheat GLK proteins were generally less conserved. However, the core Myb-DNA-binding domain itself exhibits a typical helix-loop-helix (HLH) structure. Notably, sequence variation was observed in the first helix within this domain, including motifs such as PELHRR, VELHRK, and EELHRQ. These variations may reflect subfunctionalization affecting DNA-binding affinity.

### Phylogenetic analysis of the GLK family

**Fig. 1:**
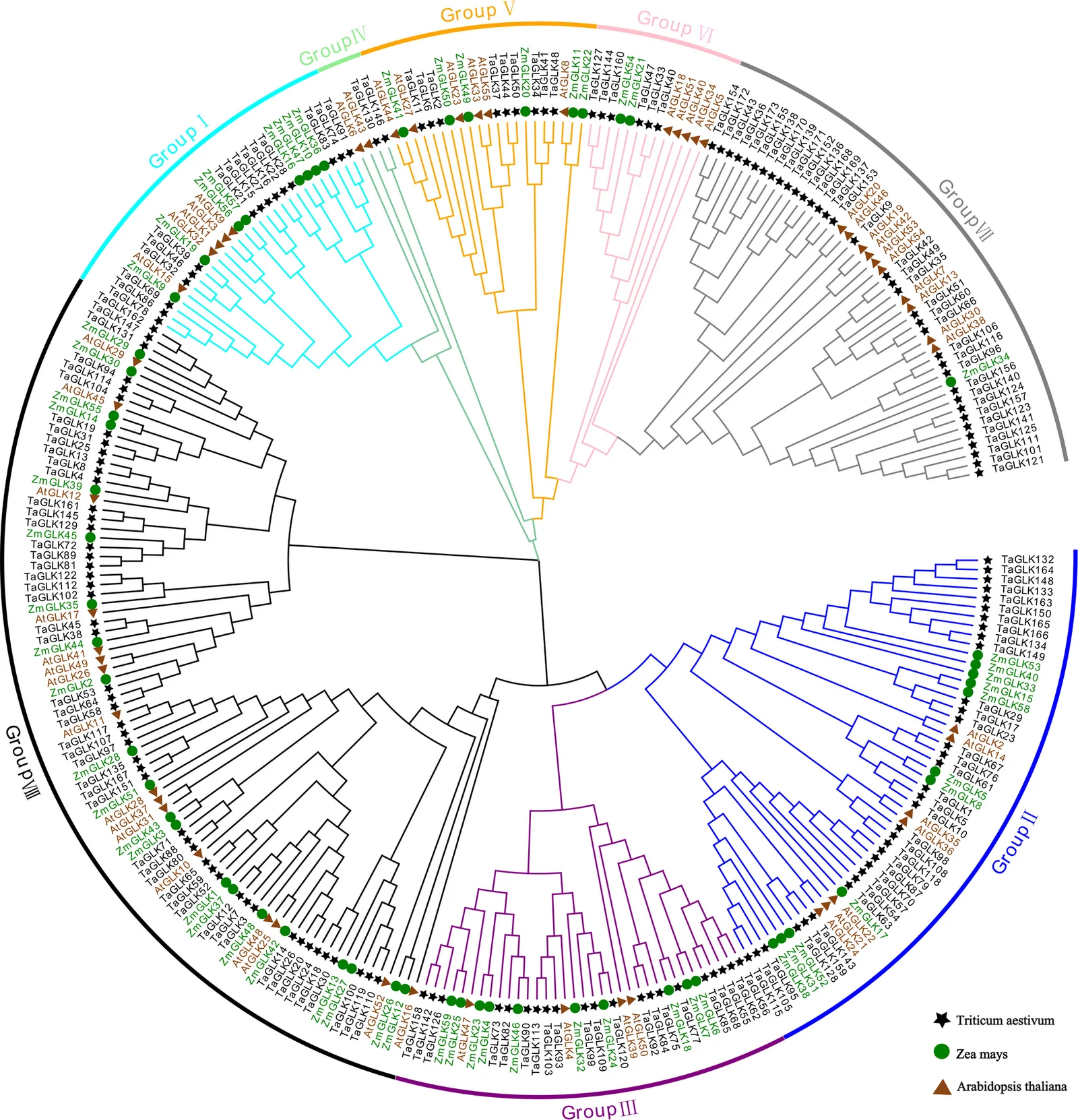
Phylogenetic tree of GLK proteins using the neighbor-joining method. Different groups were distinguished by the outer ring colors and branch colors. The proteins of wheat (*Triticum aestivum* L.; Ta), Maize (*Zea mays* L.; Zm), and *A. thaliana* (*Arabidopsis thaliana* L.; At), were displayed with black stars, green circle and SaddleBrown triangle in the inner circle

A phylogenetic analysis was conducted using 173, 55, and 59 GLK proteins from wheat, *A thaliana*, and maize, respectively (Fig. 1 and Table S2). The resulting tree revealed that subfamily VIII contained the largest number of *GLK* members (87), of which 50 were from wheat, 21 were from maize, and 16 were from *A thaliana*. In contrast, Group IV was the smallest, containing only four members, of which wheat contributed the fewest. Considering the allohexaploid nature of wheats, *GLK* genes were distributed relatively uniformly across their A, B, and D subgenomes, typically appearing as sets of three homologous copies (Fig. 1) [44, 45]. For instance, *TaGLK97*, *TaGLK107*, and *TaGLK117* from subfamily VIII constituted one such homologous trio. The clustering of TaGLKs with maize homologs in subfamily VIII suggests potential conservation of light and Pi signaling roles. These findings confirmed that a significant proportion of the *GLK* family in wheat existed as triplicate homeologs derived from its subgenomes.

### Chromosomal Localization Analysis of the *TaGLK* Gene Family

**Fig. 2:**
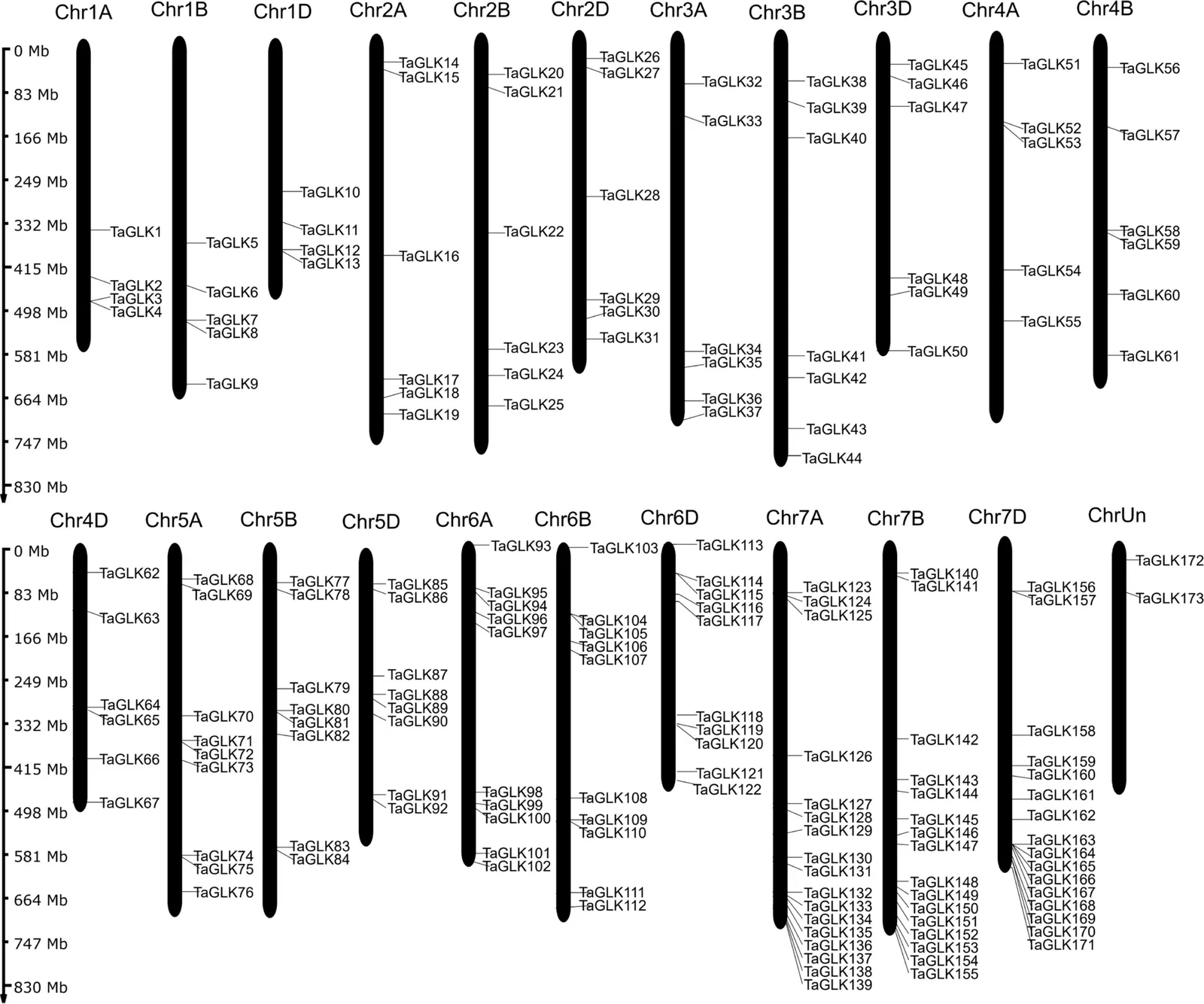
Localization of wheat *GLK* genes on chromosomes

The 173 *TaGLK* genes were distributed across all wheat chromosomes, at a variable density ranging from 2 to 16 genes per chromosome (Fig. 2). The majority were located on the homologous chromosomes 5, 6, and 7, suggesting​ that these groups underwent large-scale gene duplication events followed by the retention of these gene copies.

### Analysis of phylogenetic, motifs and gene structures of TaGLK family members

**Fig. 3:**
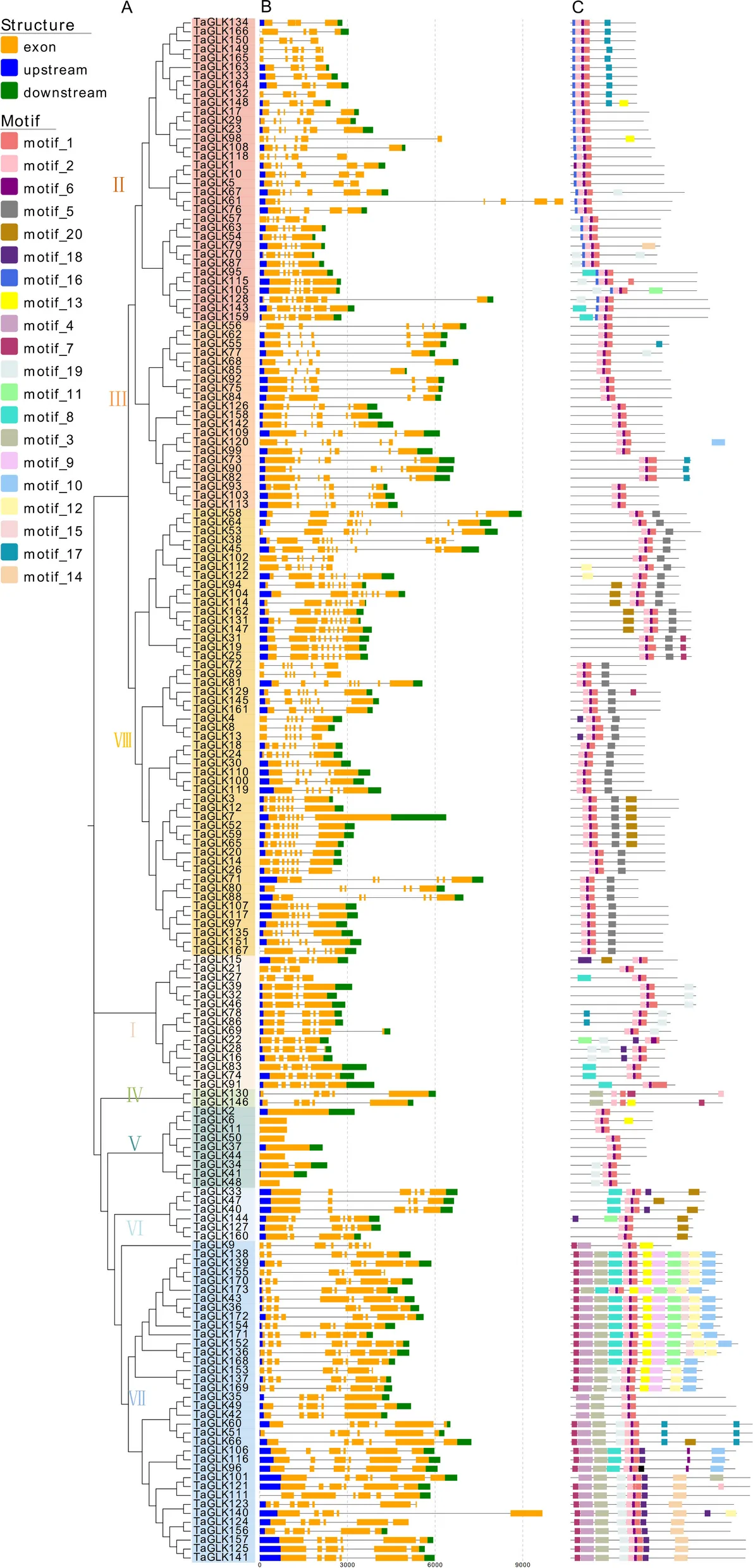
Phylogenetic evolutionary tree-based gene structure and conserved motif analysis of the TaGLK Family. **A** Different groups in the phylogenetic tree were distinguished by inconsistent colors. **B** The gene structure corresponded with the specified sequence length. The 5’ UTR, exons, and 3’ UTR were visually denoted by boxes in blue, orange, and green, respectively. **C** Conserved motifs corresponded to the sequence of protein sequences

The phylogenetic analysis revealed that TaGLK proteins within the same subfamily had similar motif compositions and arrangements. This structural conservation aligned with their evolutionary relationships and suggested that they had similar biological functions. Through an integrated analysis of phylogeny, gene structure, and motifs, 6 potential motifs were identified in the TaGLK family: Myb_SHAQKYF, PLN03162, REC superfamily, REC_typeB_ARR-like, and SANT superfamily. The majority of the 173 TaGLK members contained the characteristic N-terminal Myb DBD and a C-terminal Myb-CC-LHEQLE domain (Table S3). All members possessed motifs 1 and 2, which together form the core HLH structure of the Myb DBD. It was possible that these motifs played a crucial role in TaGLK function. Gene structure analysis revealed significant diversity, with exon numbers ranging from 1 to 10 (Fig. 3 and Table S4). Members of Subfamily V possessed the fewest exons, and some genes lacked UTRs entirely. Furthermore, motif composition varies distinctly among subfamilies (Table S5). For example, subfamily VI was characterized by motifs 1, 2, 6, and 20, whereas subfamily VIII was defined by motifs 1, 2, 5, and 6. Subfamily-specific motif enrichment suggested​ functional diversification.

### Genomic duplication drives TaGLK expansion

**Fig. 4:**
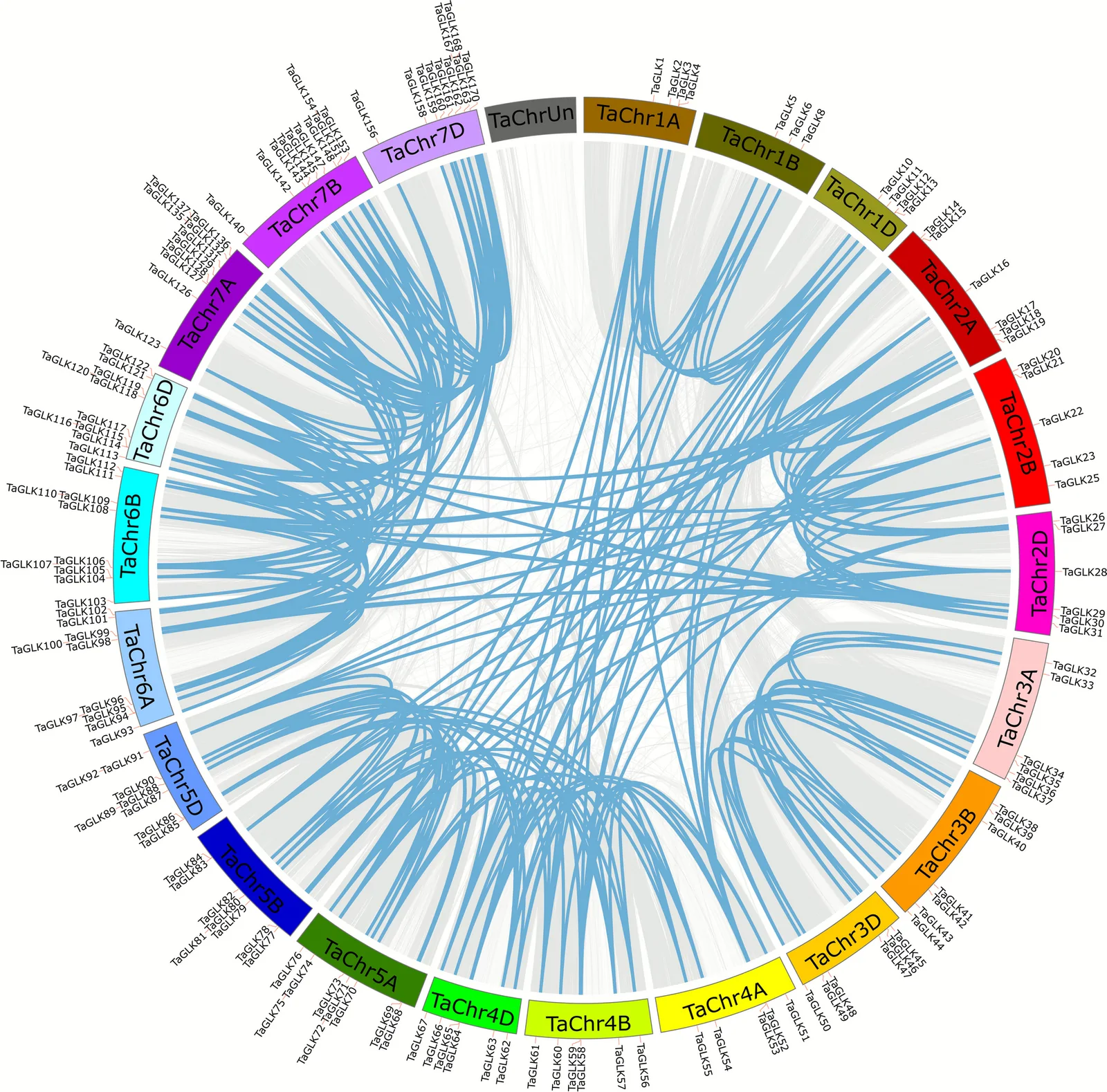
Analysis of duplication events of *GLK* genes in wheat. The gray lines in the background represented the segmental duplication in the whole *T. aestivium* genome. Blue lines represent *TaGLK* gene pairs with segmental duplication. The outer ring represents different chromosomes in the *T. aestivium* genome

The expansion of the *TaGLK* gene family was investigated through collinearity analysis, which identified 229 duplicated gene pairs within the wheat genome (Fig. 4 and Table S6). These pairs were predominantly located on chromosomes 2 A, 5D, 6D, 7 A, and 7D, and all were classified as segmental duplications, identifying this as the principal mechanism for the family''s expansion. To understand the evolutionary constraints on these genes, we calculated their Ka/Ks ratios. The results showed that all values were less than 1 (with most below 0.5), indicating that the TaGLK family could have been subjected to strong purifying selection (Table S6).

**Fig. 5:**
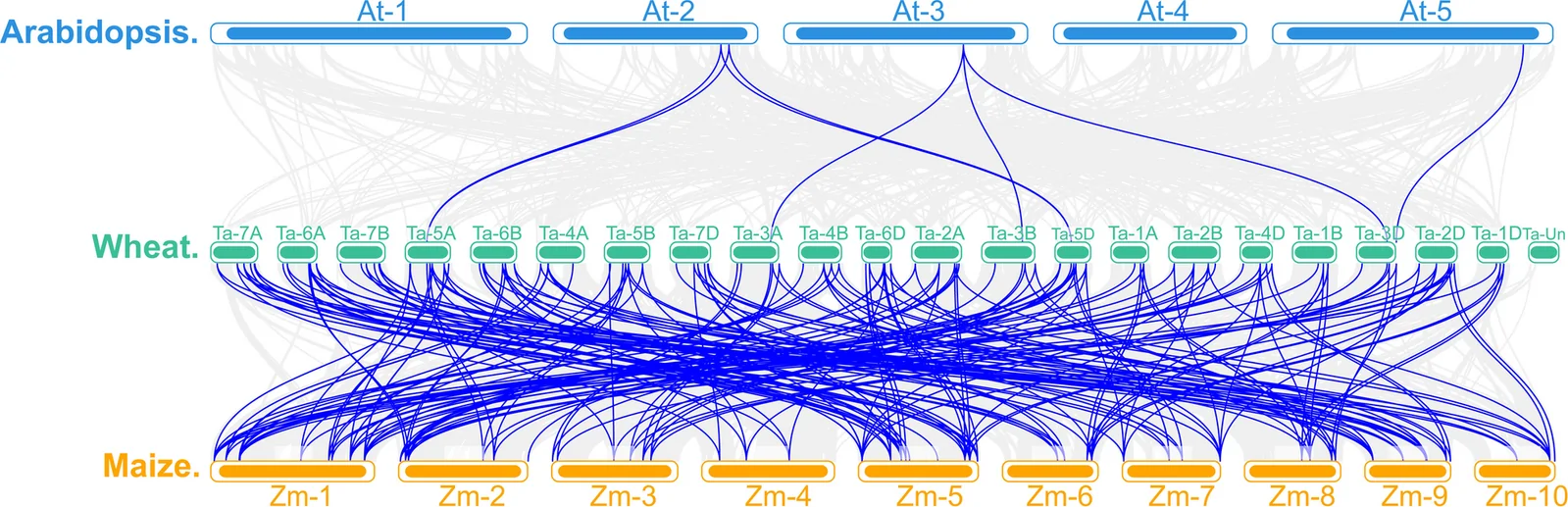
Collinearity analysis of the *GLK* gene among different species. The chromosomes of *A. thaliana*, wheat and Maize were presented in light blue, light green and light orange boxes respectively. Inter-genomic and inter-species collinearity were depicted by gray and blue lines, respectively

Synteny analysis revealed limited conservation between wheat and *A thaliana*, with only 6 *TaGLK* genes forming 8 collinear pairs (Fig. 5). By contrast, a high degree of collinearity was observed with maize, involving 212 gene pairs from 114 *TaGLK* genes, which were often present as large conserved segments. These homologues offer clues about TaGLKs protein function. The following is a list of studies on the functions of TaGLK proteins homologues in maize and *A thaliana*, identified through homologs analysis (Table 1).

**Table 1:** *TaGLK* collinearity analysis homologous gene function table

Name	Homologous gene ID	Function	Reference
*TaGLK70/87*	AT2G40260.1/AT2G38300.1	Regulators in drought tolerance and wax biosynthesis	Liu et al. [46]
*TaGLK73/84/90*	Zm00001eb041890_T005	Related to the Abaxial/Adaxial Polarity of Sheath Tissues	Héctor et al. [47]
*TaGLK33/40*	Zm00001eb118900_T002	Related to chloroplast development and photosynthesis	Rossini et al. [10, 48]
*TaGLK22/28*	Zm00001eb298990_T001	Promote the transcription of cytokinin-responsive genes	Qi et al. [49]
*TaGLK135/151/167*	Zm00001eb389770_T001	Promote the transcription of cytokinin-responsive genes	Qi et al. [49]

### Conserved TaGLK interaction modules reveal stress and developmental functions

**Fig. 6:**
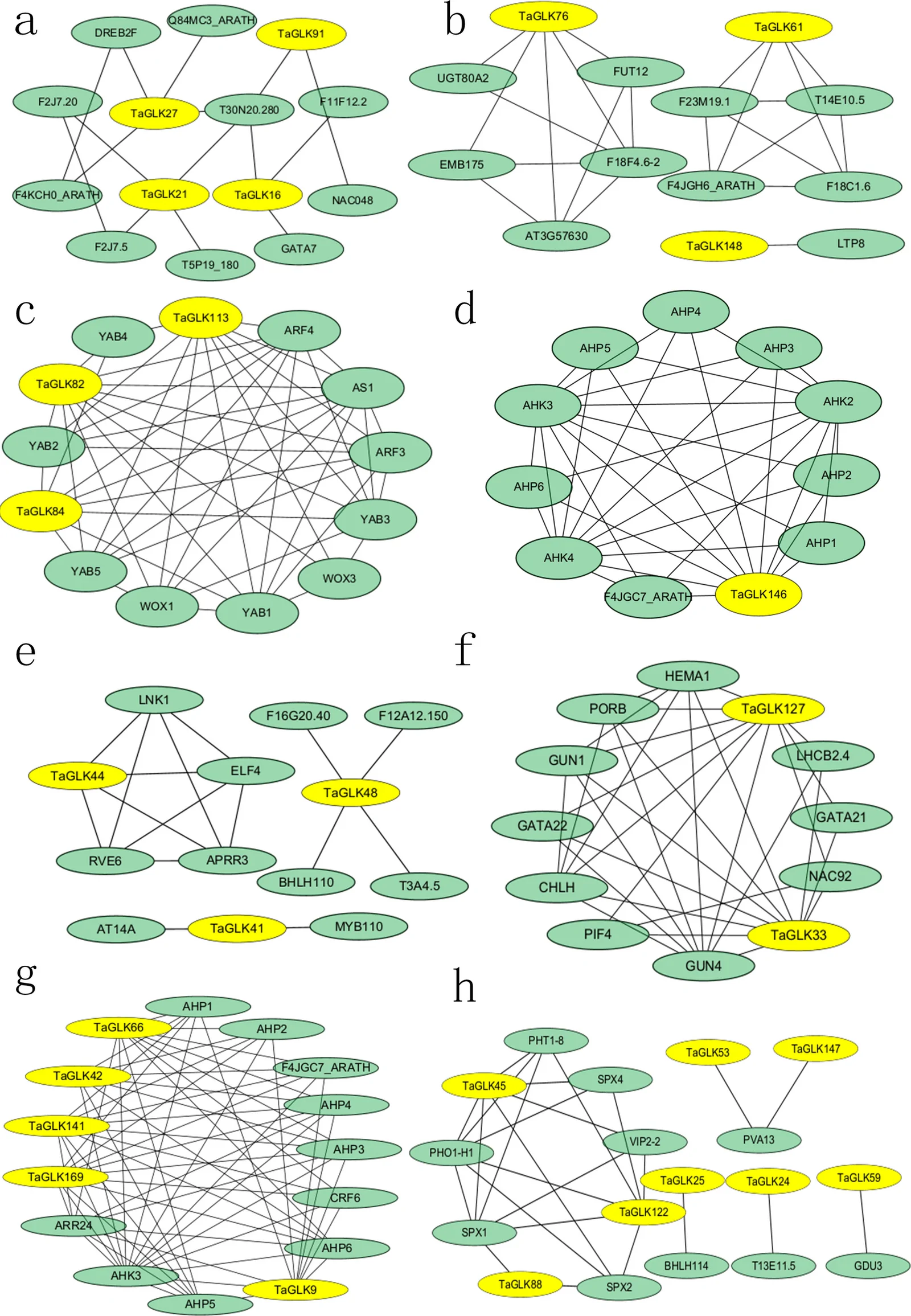
Interaction network between TaGLK proteins and other proteins. The protein interaction networks for groups I-VIII were shown in Fig. 6a-h, respectively

By using the STRING database, we created a network of predicted protein–protein interactions for TaGLK proteins based on their *A. thaliana* homologues (Fig. 6a-h and Table S7). The results suggested that the subfamilies have had distinct functional roles. Subfamily III appeared to play a crucial role in growth and development, regulating processes such as cell differentiation, embryonic development, floral organ formation, and auxin-related gene expression. Key members of this subfamily, such as TaGLK82, TaGLK84, and TaGLK113, were predicted to interact with regulators including YABBY family proteins (YAB1–5), auxin response factors (ARF3/4), and WUSCHEL-related homeobox proteins (WOX1/3). Subfamilies IV and VII were involved in auxin signaling and meristem establishment. For example, TaGLK9, TaGLK42, and TaGLK66 were predicted to interact with Histidine-containing phosphotransfer protein 1–6 (AHP1–6), and the histidine kinase AHK3, and TaGLK146 could interact with AHK2/4. Several members, including TaGLK42 and TaGLK66, also showed potential to interact with ARR24. In contrast, subfamily VI might be associated with the development of chloroplasts and photosynthesis. It acted as a nuclear gene activator for the biosynthesis of chlorophyll, the assembly of light-harvesting complexes, and the transport of electrons. For example, TaGLK33 and TaGLK127 were predicted to interact with key chloroplast-related proteins such as HEMA1, CHLH, and GUN1, as well as light-responsive proteins such as LHCB2.4 and PIF4. Subfamily VIII appeared to be prominently associated with the Pi starvation response. A major subgroup, comprising TaGLK45 and TaGLK122, was predicted to interact with SPX domain proteins (SPX1, SPX2, and SPX4), which modulate the activity of PHR1. These TaGLKs also interacted with PHO1-H1 and the Pi transporter PHT1-8, suggesting they likely played a coordinated role in Pi homeostasis. In summary, the predicted interaction network indicated that TaGLK proteins played a vital role in regulating plant growth and development, light response, and chlorophyll synthesis, and in mediating adaptation to Pi deficiency. These computational predictions provide a valuable foundation for subsequent experimental validation.

### Promoter analysis of *TaGLK*

**Fig. 7:**
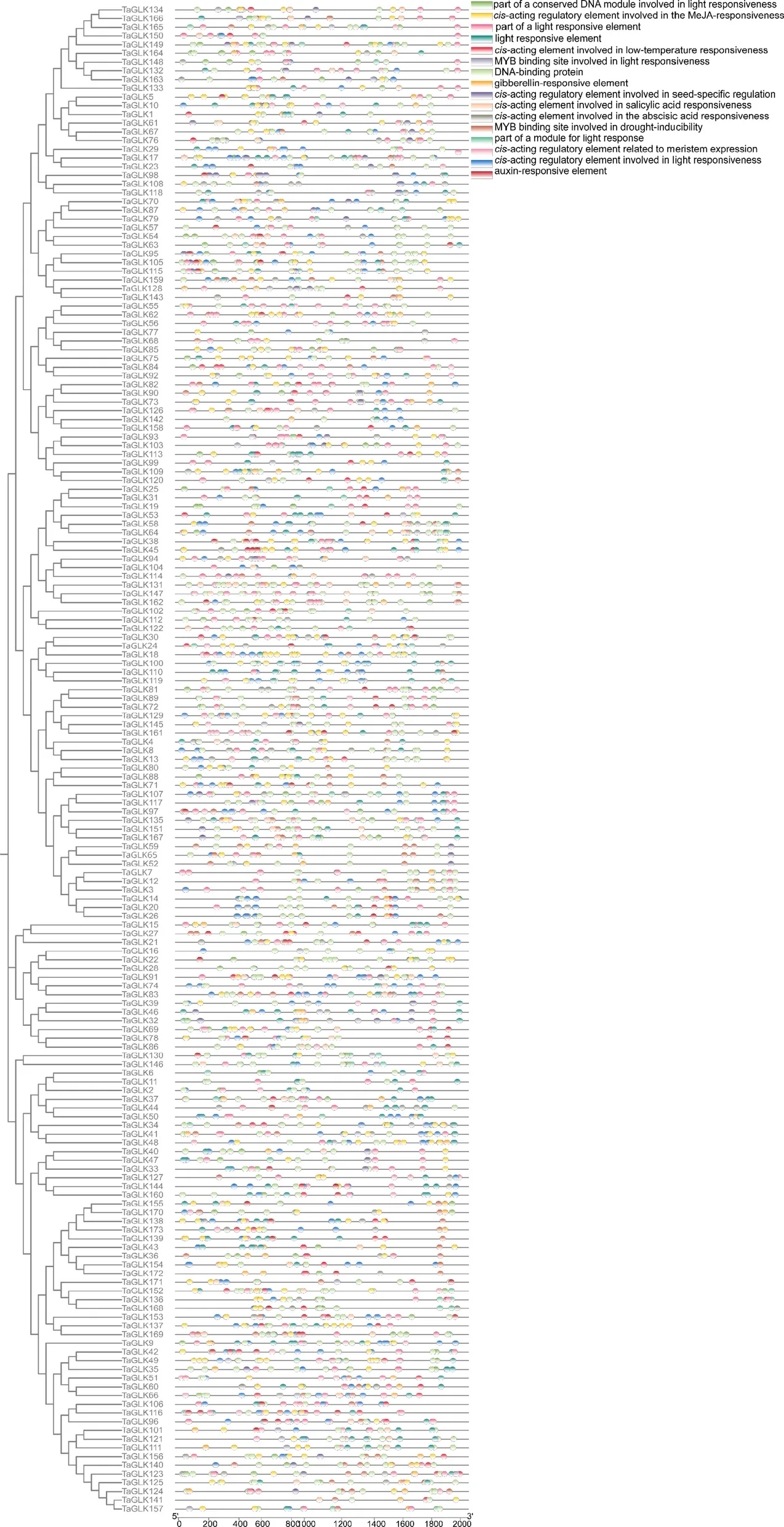
Promoter analysis of the *TaGLK* genes. The lengths and positions of different types of *cis*-acting elements correspond to the DNA sequence fragments displayed at the bottom

The *cis*-acting elements predicted in the *TaGLK* promoters were categorized into four major groups based on their regulatory functions (Fig. 7 and Table S8). These include elements associated with plant growth and development (e.g., the CAT box and RY elements), the response to abiotic stress (e.g., the LTR and MBS elements), phytohormone signaling (e.g., the ABRE and CGTCA/TGACG elements), and light responsiveness (e.g., the Box 4, GATA motif, and G-box elements). A notable variation in the repertoire and quantity of these elements was observed across the different *TaGLK* gene promoters.

The most abundant categories within the *TaGLK* promoter architecture were light- and hormone-responsive elements (Fig. 7 and Table S8). A diverse array of light-responsive elements was identified, including G-boxes, GATA motif, SP1 motif, Box 4 motif, TCT motif, GT1 motif, TCCC motif, I-boxes, AE-boxes, and MREs. Some promoters, such as those of *TaGLK9*, *TaGLK49*, and *TaGLK82*, contained up to 16 major light-responsive elements. Furthermore, nearly all *TaGLK* genes contained hormone-responsive elements (HREs). The most common of these was the abscisic acid (ABA)-responsive element (ABRE), which was present in 90.17% (156 out of 173) of the *TaGLK* genes examined. Promoters also frequently contained elements responsive to methyl jasmonate (MeJA; CGTCA and TGACG element), salicylic acid (SA; TCA element), and gibberellin (GARE motif). Additionally, the presence of the MBS element, which binds MYB transcription factors, indicated a potential regulatory association with responses to drought and salinity stress.

### miRNA prediction of* TaGLK*

The analysis revealed that the *TaGLK* genes were targeted by 45 distinct miRNAs, including Tae-mir1122a, Tae-mir1122c-3p, and Tae-mir1129 (Fig. S2 and Table S9). A Cytoscape network visualization of these interactions showed that a single miRNA can target multiple *TaGLK* genes. For instance, Tae-mir399 targets *TaGLK96*, *TaGLK106*, and *TaGLK116*, whereas Tae-mir9676-5p targets *TaGLK33*, *TaGLK35*, *TaGLK40*, *TaGLK42*, *TaGLK49*, and *TaGLK149*. Interestingly, Tae-mir9780 was predicted to have two distinct targeting relationships with *TaGLK31*. Further analysis showed that *TaGLK31* produces two splicing variants, *TaGLK31-001* and *TaGLK31-002*, which possessed different first exons (Fig. S3 and Table S9). This indicated that the two miRNA target sites were probably located on these distinct exons, enabling miR9780 to potentially regulate the abundance of each variant independently. These results provided preliminary predictions that require further experimental validation in wheat.

### Tissue-specific expression

**Fig. 8:**
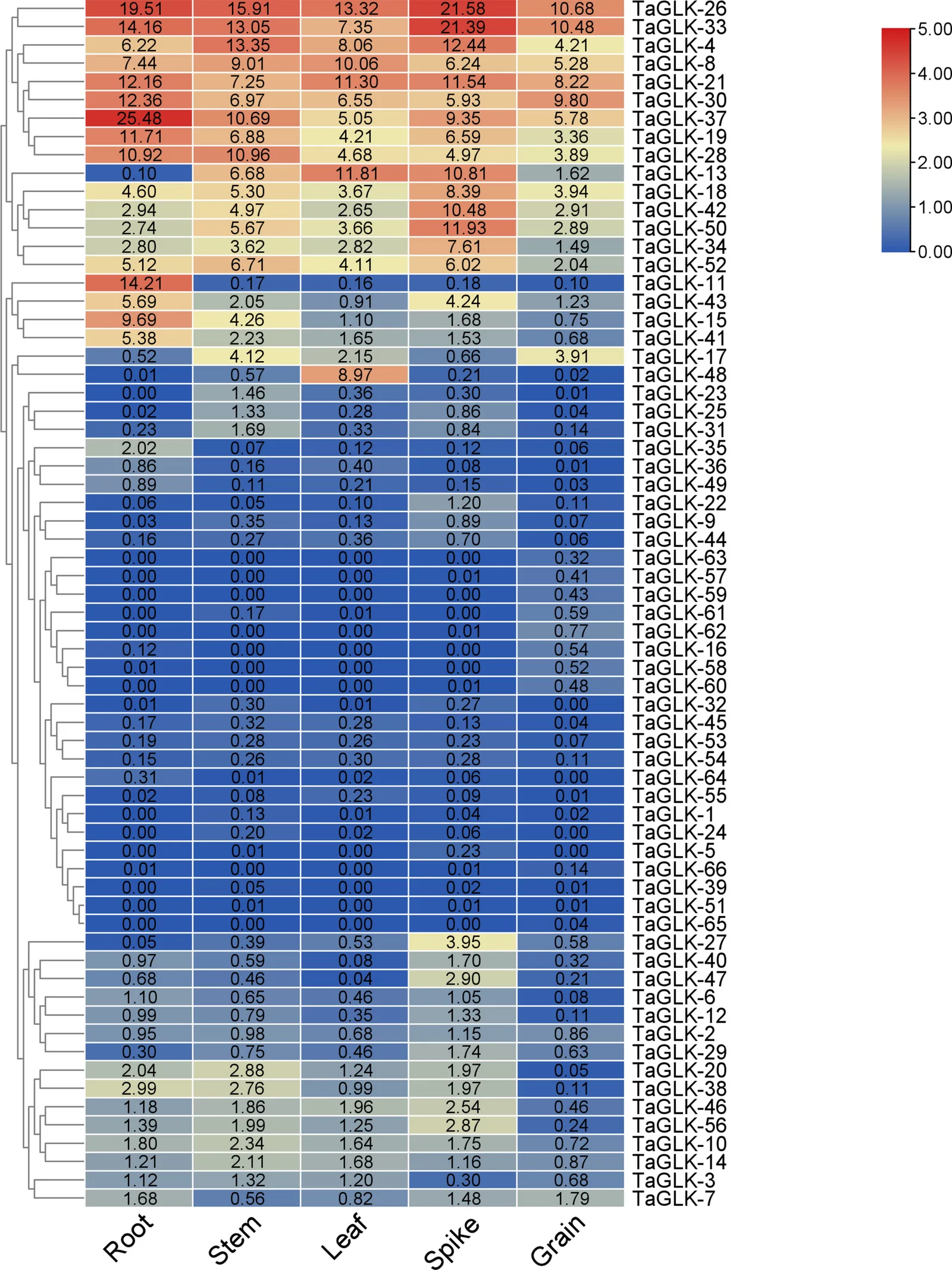
Expression of wheat *GLK* genes during different tissues. The heatmap was generated based on log2 (TPM + 1) expression values. The **A**, **B** and **D** subgenomes were calculated and utilized [50]

The analysis of RNA-seq data revealed significant variation in the expression of *TaGLK* genes in various tissues, such as roots, stems, leaves, spikes, and grains (Fig. 8 and Table S10). Transcript levels ranged from 1 to 25.48 TPM, with most genes showing low expression. Expression patterns fell into three broad categories: constitutive expression in all tissues, tissue-specific expression, and no detectable expression (TPM < 1). For example, 14 genes, including *TaGLK-4*, *TaGLK-8*, and *TaGLK-18*, exhibited constitutive expression in all tissues. In contrast, others, such as *TaGLK-11* and *TaGLK-35*, were only detected in roots, while *TaGLK-23*, *TaGLK-25*, and *TaGLK-31* were stem-specific. The genes with relatively high expression were predominantly found in subfamilies VI and VIII. These subfamilies were characterized by unique motifs: motif_20 in subfamily VI and motif_5 in subfamily VIII (Fig. 3). This finding implied a potential link between these structural features and elevated expression. A key example was TaGLK-26, which exhibited high expression (TPM > 10) across all five tissues. Overall, these results demonstrated that *TaGLK* genes exhibited significant differences in expression levels and high tissue specificity.

### light/dark response

**Fig. 9:**
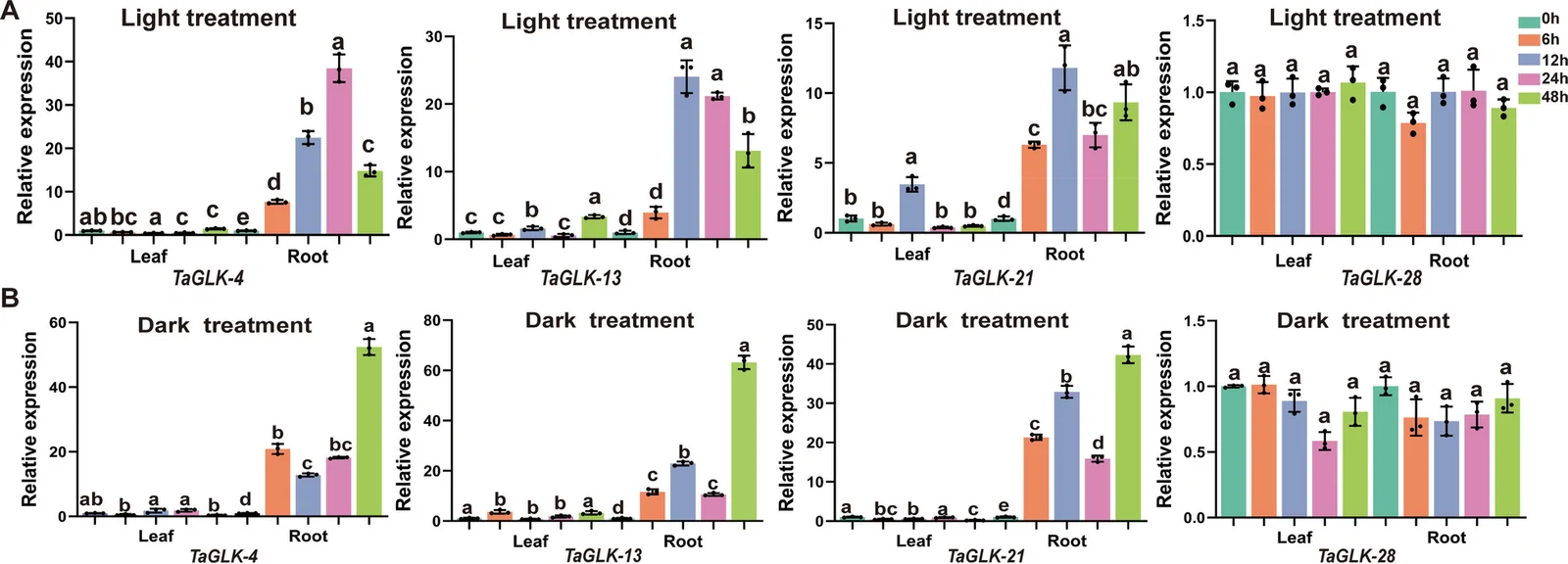
Expression pattern analysis of *GLK* genes in wheat under Light and Dark Treatments. **A** Light treatment of 12000 lx, (**B**) Dark treatment, Different letters above the bar chart indicated significant differences between different treatments (*p* < 0.05)

The expression of these four genes was validated under four different conditions. The expression patterns of these four genes varied significantly across the treatments. Following a 48-h light treatment, the transcript levels of *TaGLK-13* in the leaves increased by 2.32-fold. A more pronounced response was observed in the roots, where the abundance of transcripts for *TaGLK-4*, *TaGLK-13*, and *TaGLK-21* increased by 21.46, 22.58, and 10.81-fold after being exposed to light for 12 h. All these changes were statistically significant (Fig. 9A). Conversely, after 12 h in the dark, the level of transcripts for *TaGLK-13* increased by 22.58-fold in the roots (Fig. 9B). Unlike the other genes studied, *TaGLK-28* expression remained unchanged in both roots and leaves under light and dark conditions. This observation suggested that it might not be directly regulated by light or darkness. In conclusion, although certain *TaGLK* genes were likely involved in wheat’s response to light, their regulation appeared to be complex and to involve signaling pathways activated by darkness.

### Abiotic stress response

RT-qPCR analysis revealed that drought stress triggered a significant upregulation of *TaGLK-4*, *TaGLK-13*, *TaGLK-21*, and *TaGLK-28* in root tissues. Their transcript levels increased by 10.60, 34.05, 8.11and 6.35-fold within 12 h (p < 0.05). Under drought treatment, the differences in transcript levels were less pronounced in leaves than in roots. The findings indicated that *TaGLK* genes could play a role in how plants respond at the transcriptional level to drought stress.

**Fig. 10:**
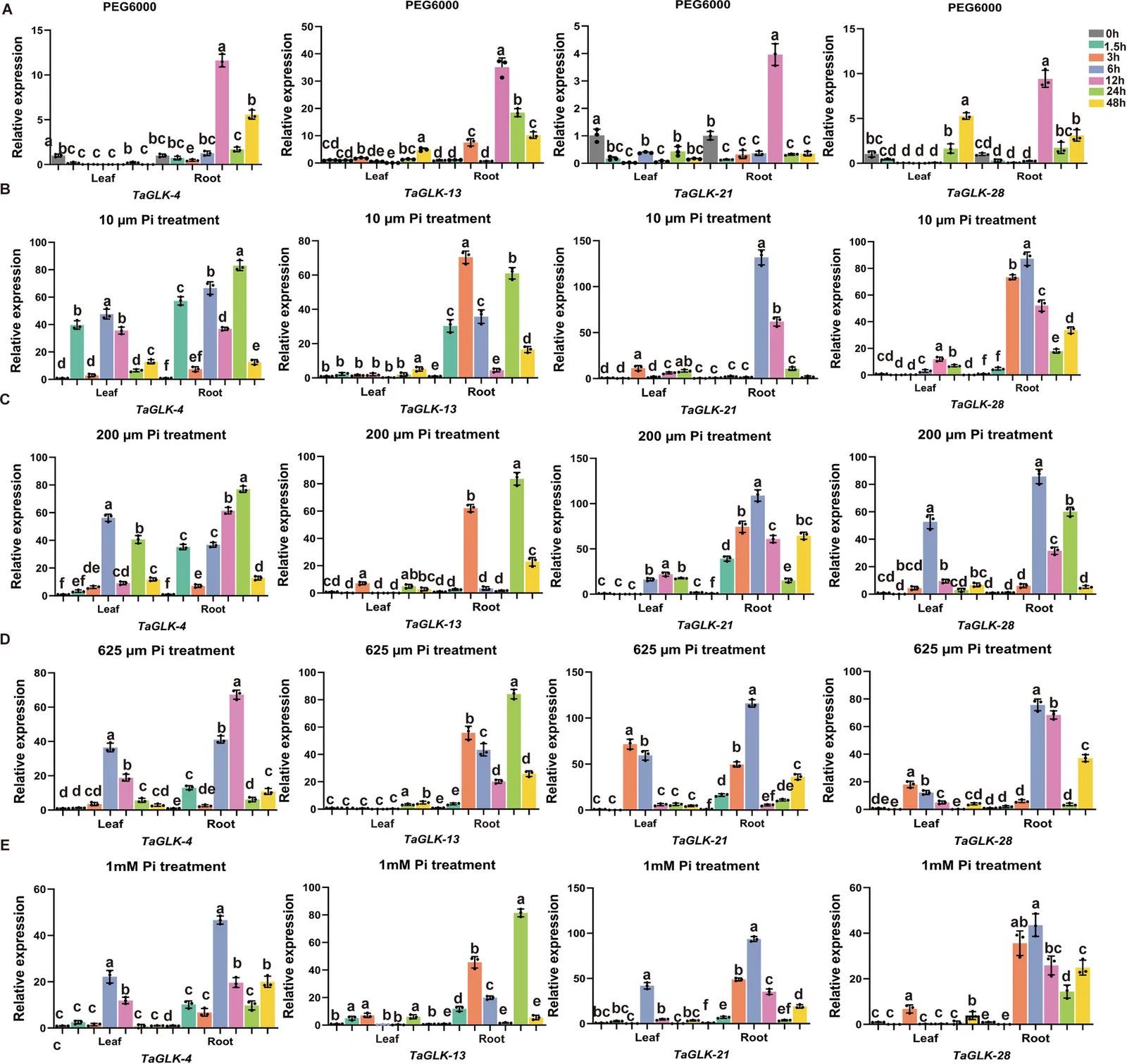
Expression Patterns in Response to Drought and Different Pi Levels. **A** 20% (w/v) PEG 6000, (**B**)10 µm Pi, (**C**) 200 µm Pi, (**D**)625 µm Pi and (**E**)1 mM Pi based on RT-qPCR. Different lowercase letters indicated significant differences (*p* < 0.05) between various time points within the same treatment

As shown in Fig. 10B-E, different Pi treatments significantly altered the expression of the *TaGLK* gene in both leaf and root tissues, with the most pronounced changes observed in the roots. Under LP conditions, the transcript levels of *TaGLK-4*, *TaGLK-21*, and *TaGLK-28* were significantly higher in the roots than in the NP and HP treatments. Specifically, 6 h after the LP treatment, the expression of *TaGLK-4* increased 65.46-fold in the roots and 46.51-fold in the leaves. Meanwhile, the expression of *TaGLK-13* and *TaGLK-28* increased 34.78- and 86.25-fold, respectively, in the roots. In contrast, the expression profiles of these four genes were similar under the two HP treatments. The findings indicated that the expression of the *TaGLK* gene might be specifically influenced by the availability of Pi and could be linked to the Pi stress signaling pathway.

### Subcellular localization of TaGLK40 and TaGLK144 proteins

The precise intracellular localization of proteins establishes specialized microenvironments that were essential for their optimal function. This localization also conferred the exact spatial coordinates that were necessary for accurately executing their biological roles. Subcellular localization experiments revealed that the fluorescent signal from the empty vector-which served as a negative control-exhibited a diffuse distribution across multiple cellular compartments, including the nucleus, cell membrane, and cytoplasm. The fluorescent signals from TaGLK40-GFP and TaGLK144-GFP were specifically localized to the nucleus. In summary, TaGLK40 and TaGLK144 demonstrated distinct nuclear localization properties that align precisely with transcription factor characteristics. These results corroborate the predicted subcellular localization outcomes.

## Discussion

### Evolutionary diversification of *TaGLK* family

A comparative analysis revealed that wheat possesses 173 *GLK* genes, significantly more than the 130 found in soybean (*Glycine max*) [51], the 78 found in moso bamboo (*Phyllostachys edulis*) [52], and the 9 found in the tea plant [53]. It is likely that this expansion in wheat was attributable to its allohexaploid nature, which resulted from two independent hybridization events between three diploid progenitors and produced a large, complex genome prone to duplication [54, 55]. *TaGLK* primarily existed as a segmental duplication (Retain sequences with length ≥ 1 kb and identity ≥ 90%, the gene pairs within the *TaGLK* genes were all located on different chromosomes) (Fig. 4 and Table S5), rather than tandem amplification, it suggested that ancient chromosomal rearrangements were the main driver of *TaGLK* expansion, which was consistent with polyploidization events. Furthermore, a protein structure analysis revealed conserved exon–intron arrangements within subfamilies, a pattern that was consistent with GLK homologues in *A thaliana* and maize [12, 14]. The GLK proteins contained 2 core conserved motifs, motif_1 and motif_2, indicating a high degree of structural conservation, as seen in other species like tea plants [56]. However, wheat exhibited greater diversity, with 7 distinct variants of Motif 1 compared to 1 to 4 in other species [9, 56]. This variation may alter DNA-binding affinity and contribute to functional diversification. An analysis of evolutionary pressure indicated that the *TaGLK* gene family had mainly been subject to purifying selection, as indicated by all Ka/Ks ratios being less than 1. This suggested that these genes might play crucial regulatory roles in photosynthesis and stress responses. Additionally, there was a greater similarity between gene pairs in wheat and maize compared to those in dicot species (Fig. 5), which could illustrate the divergent evolutionary trajectories that the GLK family underwent in monocots versus dicots.

### Functional specialization under light

The *GLK* family of transcription factors was well-established in the regulation of chloroplast development and chlorophyll biosynthesis, which ultimately influenced photosynthetic efficiency and crop yield [19, 57–59]. Subfamily VI TaGLKs (such as TaGLK-13) acted as core regulators of chloroplast development and photosynthetic gene expression, similar to GLK1/2 in *A thaliana* and ZmG2 [10, 58]. Conservation of light-responsive motif (G-box) in subfamily VI TaGLKs, which was a known light-responsive motif that recruited transcription factors to activate chlorophyll biosynthesis [60]. Interaction of TaGLKs with light signaling proteins (HY5, PIF4) were Predicted,

which could serve as a reference for studying the function of the TaGLK protein gene in wheat.

The localization of proteins TaGLK40 and TaGLK144 in the nucleus was consistented with previous findings, indicating their nuclear localization properties [8]. In summary, GLKs acted as central regulatory nodes that integrate light signals, chloroplast biogenesis, and stress responses, making them key targets for improving crop productivity [56, 61]. Given the central role of GLKs in light signaling, the conservation of subfamily VI TaGLKs was thought to have highlighted potential avenues for enhancing photosynthetic efficiency in wheat breeding.

### Translational implications for wheat improvement

#### Drought response via ABA-mediated and independent pathways (subfamily Ⅵ)

The drought-induced upregulation of *TaGLK-13* suggested it may function​ as a positive regulator of drought tolerance. This role was supported by evidence from other species; for instance, overexpressing *ZmGLK1* or *GOLDEN2* (*ZmG2*) in rice enhances drought tolerance by promoting ABA-mediated stomatal closure [19]. In wheat, the miRNA tae-miR9676-5p, which was strongly responsive to drought memory [62], was predicted to target *TaGLK33* and *TaGLK40*. This regulatory relationship suggested that *TaGLK33* and *TaGLK40* might also play a significant role in enhancing the plant''s drought tolerance.

#### Role of TaGLK (Subfamily VIII) in Pi Deficiency Response via SPX/PHT Interaction

**Fig. 11:**
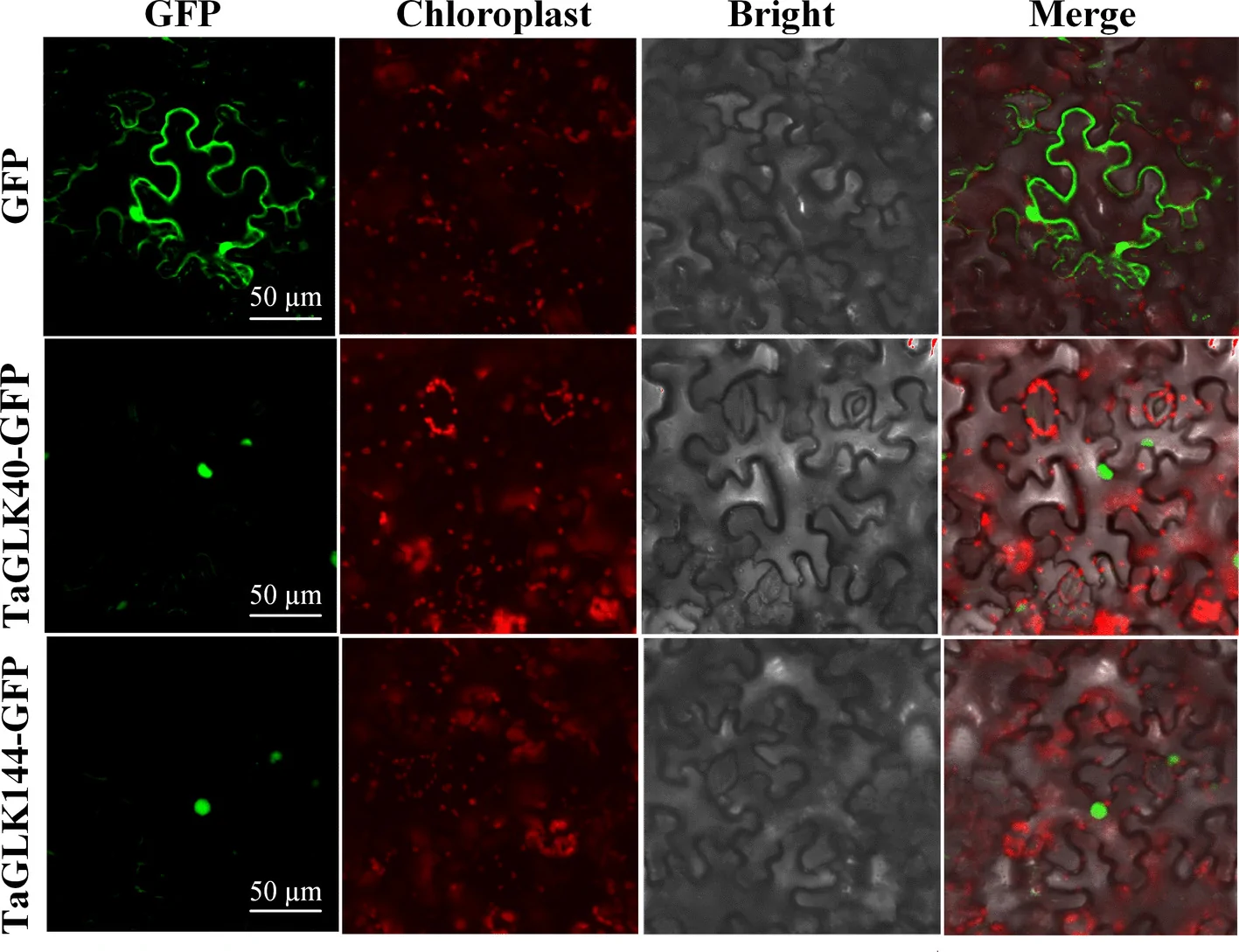
Subcellular localization of the TaGLK40 and TaGLK144 protein

The efficient absorption and utilization of Pi was vital for maximizing crop yield. Our findings suggested that specific *TaGLK* genes play a role in this process. The unique motif_5 (LHEQLE) was found in members of subfamily VIII and had previously been linked to the Pi starvation response [63, 64]. Supporting this, predictions of protein–protein interactions indicated that several TaGLK proteins (TaGLK45, TaGLK88 and TaGLK122) interacted with key components of the Pi signaling pathway, including SPX domain proteins, which were activated under Pi deficiency, and the Pi transporter PHT1-8 (Fig. 6 and Table S7) [65, 66]. The genetic evidence was corroborated by expression data. The expression of *TaGLK* genes responded differentially to varying Pi levels, with the most pronounced changes observed in root tissues (Fig. 11). In conclusion, the conserved motif, predicted protein interactions, and Pi-responsive expression patterns suggested that *TaGLK* transcription factors might be involved in the Pi starvation signaling pathway and could enhance Pi uptake in wheat.

## Conclusions

This study successfully identified 173 putative *GLK* transcription factors in the wheat genome. We systematically characterized these *TaGLK* members by analyzing their physicochemical properties, evolutionary relationships, gene structures, conserved motifs, duplication events, promoter *cis*-elements, miRNA targets, and expression patterns across tissues and under various stress treatments. Our observations suggest potential functional differentiation within the *TaGLK* family. *TaGLK* could be​ involved in chloroplast development and light/dark responses, while also potentially playing a role​ in abiotic stress responses by positively regulating drought tolerance and adaptation to low Pi stress. This in silico analysis serves as a starting point for exploring the evolution and possible roles of the *TaGLK* gene family, primarily offering speculation. To confirm and expand upon these computational insights, future research should concentrate on functional genomics and experimental biology.

## Supplementary Information

Supplementary Material 1.

Supplementary Material 2.

Supplementary Material 3.

Supplementary Material 4.

Supplementary Material 5.

Supplementary Material 6.

Supplementary Material 7.

Supplementary Material 8.

Supplementary Material 9.

Supplementary Material 10.

Supplementary Material 11.

Supplementary Material 12.

## Data Availability

The datasets supporting the results of this article are included in the article and its Additional Files.

## Abbreviations

SMART: Simple Modular Architecture Research Tool
NCBI-CDD: National Center for Biotechnology Information-Conserved Domain Database
HEMA1: Glutamyl-tRNA reductase 1
GUN1/4: Genomes uncoupled 1/4
PORB: Protochlorophyllide reductase B
GATA21/22: GATA transcription factor 21/22
CHLH: Magnesium chelatase subunit H
PIF4: Phytochrome-interacting factor 4
LHCB2.4: Chlorophyll a/b-binding protein 2.4
NAC92: NAC domain containing protein 92
HY5: Elongated hypocotyl 5
GFP: Green Fluorescent Protein
PHO1-H1: phosphate 1 homolog 1 (PH1H1, also historically known as PHO1-H1)
SPX1: SPX domain-containing protein 1
AHP1: Histidine-containing phosphotransfer protein 1
YAB1: YABBY 1
WOX1: WUSCHEL-related homeobox 1
AS1: Asymmetric leaves 1
DREB2F: Dehydration-responsive element-binding protein 2F, et al.

## References

[1] Bailey-Serres J, Parker JE, Ainsworth EA, Oldroyd GED, Schroeder JI. Genetic strategies for improving crop yields. Nature. 2019;575(7781):109–18. 10.1038/s41586-019-1679-0 PMC7024682 31695205

[2] Zhang Y, Tian L, Lu C. Chloroplast gene expression: recent advances and perspectives. Plant Commun. 2023;4(5):19. 10.1016/j.xplc.2023.100611 PMC10504595 37147800

[3] Thakro V, Malik N, Basu U, Srivastava R, Narnoliya L, Daware A, et al. A superior gene allele involved in abscisic acid signaling enhances drought tolerance and yield in chickpea. Plant Physiol. 2023;191(3):1884–912. 36477336 10.1093/plphys/kiac550 PMC10022645

[4] Ouyang X, Zhong X, Chang S, Qian Q, Zhang Y, Zhu X. Partially functional NARROW LEAF1 balances leaf photosynthesis and plant architecture for greater rice yield. Plant Physiol. 2022;189(2):772–89. 35377451 10.1093/plphys/kiac135 PMC9157069

[5] Han Y, White PJ, Cheng L. Mechanisms for improving phosphorus utilization efficiency in plants. Ann Bot. 2022;129(3):247–58. 34864840 10.1093/aob/mcab145 PMC8835619

[6] Tian MZ, Wang HF, Tian Y, Hao J, Guo HL, Chen LM, et al. *Zm*PHR1 contributes to drought resistance by modulating phosphate homeostasis in maize. Plant Biotechnol J. 2024;22(11):3085–98. 39037027 10.1111/pbi.14431 PMC11500998

[7] Fitter DW, Martin DJ, Copley MJ, Scotland RW, Langdale JA. GLK gene pairs regulate chloroplast development in diverse plant species. Plant J. 2002;31(6):713–27. 12220263 10.1046/j.1365-313x.2002.01390.x

[8] Zhang D, Tan W, Yang F, Han Q, Deng X, Guo H, et al. A BIN2-GLK1 signaling module integrates brassinosteroid and light signaling to repress chloroplast development in the dark. Dev Cell. 2021;56(3):310–24. 33357403 10.1016/j.devcel.2020.12.001

[9] Bian X, Qu C, Jiang J, Liu G. Genome-wide identification of GLK gene family and the role of BpHHO4 in birch leaf morphology. J Plant Growth Regul. 2025. 10.1007/s00344-025-11685-9.

[10] Rossini L, Cribb L, Martin DJ, Langdale JA. The maize golden2 gene defines a novel class of transcriptional regulators in plants. Plant Cell. 2001;13(5):1231–44. 11340194 10.1105/tpc.13.5.1231 PMC135554

[11] Chang Y-M, Liu W-Y, Shih AC-C, Shen M-N, Lu C-H, Lu M-YJ, et al. Characterizing regulatory and functional differentiation between maize mesophyll and bundle sheath cells by transcriptomic analysis. Plant Physiol. 2012;160(1):165–77. 22829318 10.1104/pp.112.203810 PMC3440195

[12] Liu F, Xu Y, Han G, Zhou L, Ali A, Zhu S, et al. Molecular evolution and genetic variation of G2-like transcription factor genes in maize. PLoS ONE. 2016;11(8):e0161763. 27560803 10.1371/journal.pone.0161763 PMC4999087

[13] Bhutia KL, Nongbri EL, Gympad E, Rai M, Tyagi W. In silico characterization, and expression analysis of rice golden 2-like (OsGLK) members in response to low phosphorous. Mol Biol Rep. 2020;47(4):2529–49. 32086721 10.1007/s11033-020-05337-2

[14] Alam I, Wu X, Yu Q, Ge L. Comprehensive genomic analysis of G2-like transcription factor genes and their role in development and abiotic stresses in Arabidopsis. Diversity. 2022;14(3):228.

[15] Frangedakis E, Yelina NE, Billakurthi K, Hua L, Schreier T, Dickinson PJ, et al. MYB-related transcription factors control chloroplast biogenesis. Cell. 2024;187(18):4859–76. 39047726 10.1016/j.cell.2024.06.039

[16] Frangedakis E, Yelina NE, Billakurthi K, Schreier T, Dickinson PJ, Tomaselli M, Haseloff J, Hibberd JM: Synergistic control of chloroplast biogenesis by MYB-related and Golden2-like transcription factors. bioRxiv - Plant Biology 2023. 10.1016/j.cell.2024.06.039 39047726

[17] Waters MT, Wang P, Korkaric M, Capper RG, Saunders NJ, Langdale JA. GLK transcription factors coordinate expression of the photosynthetic apparatus in Arabidopsis. Plant Cell. 2009;21(4):1109–28. 19376934 10.1105/tpc.108.065250 PMC2685620

[18] Tachibana R, Abe S, Marugami M, Yamagami A, Akema R, Ohashi T, et al. BPG4 regulates chloroplast development and homeostasis by suppressing GLK transcription factors and involving light and brassinosteroid signaling. Nat Commun. 2024;15(2):370. 38191552 10.1038/s41467-023-44492-5 PMC10774444

[19] Li X, Li J, Wei S, Gao Y, Pei H, Geng R, et al. Maize GOLDEN2-LIKE proteins enhance drought tolerance in rice by promoting stomatal closure. Plant Physiol. 2024;194(2):774–86. 37850886 10.1093/plphys/kiad561 PMC10828204

[20] Luo J, Zheng M, He J, Lou Y, Ge Q, Ma B, et al. Sunflower HaGLK enhances photosynthesis, grain yields, and stress tolerance of rice. Biology. 2025. 10.3390/biology14080946. 40906084 PMC12384029

[21] Liu J, Mehari TG, Xu Y, Umer MJ, Hou Y, Wang Y, et al. GhGLK1 a key candidate gene from GARP family enhances cold and drought stress tolerance in cotton. Front Plant Sci. 2021;12:759312. 34992618 10.3389/fpls.2021.759312 PMC8725998

[22] Liu X, Li L, Li M, Su L, Lian S, Zhang B, et al. AhGLK1 affects chlorophyll biosynthesis and photosynthesis in peanut leaves during recovery from drought. Sci Rep. 2018;8(1):2250. 29396501 10.1038/s41598-018-20542-7 PMC5796971

[23] Kobayashi K, Sasaki D, Noguchi K, Fujinuma D, Komatsu H, Kobayashi M, et al. Photosynthesis of root chloroplasts developed in Arabidopsis lines overexpressing GOLDEN2-LIKE transcription factors. Plant Cell Physiol. 2013;54(8):1365–77. 23749810 10.1093/pcp/pct086 PMC3730084

[24] Li Y, Li Y, Yao X, Wen Y, Zhou Z, Lei W, et al. Nitrogen-inducible GLK1 modulates phosphate starvation response via the PHR1-dependent pathway. New Phytol. 2022;236(5):1871–87. 36111350 10.1111/nph.18499

[25] Wu Y, Zhu H, Wang S, Liu H, Yang Q, Wang X. Genome-wide identification and expression analysis of the GARP superfamily in response to abiotic stresses in alfalfa (*Medicago sativa* L.). BMC Plant Biol. 2025. 10.1186/s12870-025-07423-8. 41107726 PMC12535029

[26] Zhang XW, An XH, Xu RR, Tian Y, Liu B, Han Y, et al. The MdGLK1-MdBZR1 module integrates ethylene and strigolactone signals to regulate cold tolerance via a CBF-dependent pathway in apple. Plant Biotechnol J. 2025. 10.1111/pbi.70325. 40824758 PMC12665089

[27] Luo X, Liu B, Xie L, Wang K, Xu D, Tian X, et al. The *TaSOC1-TaVRN1* module integrates photoperiod and vernalization signals to regulate wheat flowering. Plant Biotechnol J. 2024;22(3):635–49. 37938892 10.1111/pbi.14211 PMC10893938

[28] Harrison PW, Amode MR, Austine-Orimoloye O, Azov AG, Barba M, Barnes I, et al. Ensembl 2024. Nucleic Acids Res. 2024;52(D1):D891–99. 37953337 10.1093/nar/gkad1049 PMC10767893

[29] Matthias B, Antonina A, Laisecavalcanti F, Sararocio C, Tiago G, Emma H, et al. InterPro: the protein sequence classification resource in 2025. Nucleic Acids Res. 2024;D1:D1. 10.1093/nar/gkae1082 PMC11701551 39565202

[30] Chen C, Wu Y, Li J, Wang X, Zeng Z, Xu J, et al. TBtools-II: A “one for all, all for one” bioinformatics platform for biological big-data mining. Mol Plant. 2023;16(11):1733–42. 37740491 10.1016/j.molp.2023.09.010

[31] Wang J, Chitsaz F, Derbyshire MK, Gonzales NR, Gwadz M, Lu S, et al. The conserved domain database in 2023. Nucleic Acids Res. 2023;51(D1):D384–8. 36477806 10.1093/nar/gkac1096 PMC9825596

[32] Letunic I, Bork P. 20 years of the SMART protein domain annotation resource. Nucleic Acids Res. 2018;46(D1):D493–6. 29040681 10.1093/nar/gkx922 PMC5753352

[33] Duvaud S, Gabella C, Lisacek F, Stockinger H, Ioannidis V, Durinx C. Expasy, the Swiss bioinformatics resource portal, as designed by its users. Nucleic Acids Res. 2021;49(W1):W216–27. 33849055 10.1093/nar/gkab225 PMC8265094

[34] Savojardo C, Martelli PL, Fariselli P, Profiti G, Casadio R. BUSCA: an integrative web server to predict subcellular localization of proteins. Nucleic Acids Res. 2018;46(W1):W459–66. 29718411 10.1093/nar/gky320 PMC6031068

[35] He Z, Zhang H, Gao S, Lercher MJ, Chen W-H, Hu S. Evolview v2: an online visualization and management tool for customized and annotated phylogenetic trees. Nucleic Acids Res. 2016;44(W1):W236–41. 27131786 10.1093/nar/gkw370 PMC4987921

[36] Chao J, Li Z, Sun Y, Aluko OO, Wu X, Wang Q, et al. MG2C: a user-friendly online tool for drawing genetic maps. Mol Hortic. 2021;1(1):16. 37789491 10.1186/s43897-021-00020-x PMC10514940

[37] Bailey TL, Johnson J, Grant CE, Noble WS. The MEME Suite. Nucleic Acids Res. 2015;43(W1):W39–49. 25953851 10.1093/nar/gkv416 PMC4489269

[38] Damian S, Katerina N, Mikaela K, Rebecca K, Farrokh M, Radja H, et al. The STRING database in 2025: protein networks with directionality of regulation. Nucleic Acids Res. 2024;D1:D1. 10.1093/nar/gkae1113 PMC11701646 39558183

[39] Shannon P, Markiel A, Ozier O, Baliga NS, Wang JT, Ramage D, et al. Cytoscape: a software environment for integrated models of biomolecular interaction networks. Genome Res. 2003;13(11):2498–504. 14597658 10.1101/gr.1239303 PMC403769

[40] Lescot M, Déhais P, Thijs G, Marchal K, Moreau Y, Van de Peer Y, et al. PlantCARE, a database of plant cis-acting regulatory elements and a portal to tools for in silico analysis of promoter sequences. Nucleic Acids Res. 2002;30(1):325–7. 11752327 10.1093/nar/30.1.325 PMC99092

[41] Dai X, Zhuang Z, Zhao PX. PsRNAtarget: a plant small RNA target analysis server (2017 release). Nucleic Acids Res. 2018;46(W1):W49–54. 29718424 10.1093/nar/gky316 PMC6030838

[42] Hu B, Jin J, Guo AY, Zhang H, Gao G. GSDS 2.0: an upgraded gene feature visualization server. Bioinformatics. 2014;31(8):1296. 25504850 10.1093/bioinformatics/btu817 PMC4393523

[43] Ma S, Wang M, Wu J, Guo W, Chen Y, Li G, et al. Wheatomics: a platform combining multiple omics data to accelerate functional genomics studies in wheat. Mol Plant. 2021;14(12):1965–8. 34715393 10.1016/j.molp.2021.10.006

[44] Guo Y, Chen Y, Wang Y, Wu X, Zhang X, Mao W, et al. The translational landscape of bread wheat during grain development. Plant Cell. 2023;35(6):1848–67. 36905284 10.1093/plcell/koad075 PMC10226598

[45] Laugerotte J, Baumann U, Sourdille P. Genetic control of compatibility in crosses between wheat and its wild or cultivated relatives. Plant Biotechnol J. 2022;20(5):812–32. 35114064 10.1111/pbi.13784 PMC9055826

[46] Liu Q, Huang H, Chen Y, Yue Z, Wang Z, Qu T, et al. Two Arabidopsis MYB-SHAQKYF transcription repressors regulate leaf wax biosynthesis via transcriptional suppression on DEWAX. New Phytol. 2022;236(6):2115–30. 36110041 10.1111/nph.18498

[47] Candela H, Johnston R, Gerhold A, Foster T, Hake S. The milkweed pod1 gene encodes a KANADI protein that is required for abaxial/adaxial patterning in maize leaves. Plant Cell. 2008;20(8):2073–87. 18757553 10.1105/tpc.108.059709 PMC2553616

[48] Cribb L, Hall LN, Langdale JA. Four mutant alleles elucidate the role of the G2 protein in the development of C4 and C3 photosynthesizing maize tissues. Genetics. 2001;159(2):787–97. 11606553 10.1093/genetics/159.2.787 PMC1461819

[49] Qi X, Zhuang Z, Ji X, Bian J, Peng Y. The mechanism of exogenous salicylic acid and 6-benzylaminopurine regulating the elongation of maize mesocotyl. Int J Mol Sci. 2024;25(11):6150. 38892338 10.3390/ijms25116150 PMC11172663

[50] Ramírez-González RH, Borrill P, Lang D, Harrington SA, Brinton J, Venturini L, et al. The transcriptional landscape of polyploid wheat. Science. 2018. 10.1126/science.aar6089. 30115782

[51] Alam I, Manghwar H, Zhang H, Yu Q, Ge L. Identification of GOLDEN2-like transcription factor genes in soybeans and their role in regulating plant development and metal ion stresses. Front Plant Sci. 2022;13:1052659. 36438095 10.3389/fpls.2022.1052659 PMC9691782

[52] Wu R, Guo L, Wang R, Zhang Q, Yao H. Genome-wide identification and characterization of G2-like transcription factor genes in moso bamboo (*Phyllostachys edulis*). Molecules. 2022. 10.3390/molecules27175491. 36080259 PMC9457811

[53] Yue C, Chen Q, Hu J, Li C, Luo L, Zeng L. Genome-wide identification and characterization of GARP transcription factor gene family members reveal their diverse functions in tea plant (*Camellia **sinensis*). Front Plant Sci. 2022;13:947072. 35845671 10.3389/fpls.2022.947072 PMC9280663

[54] Marcussen T, Sandve SR, Heier L, Spannagl M, Pfeifer M, Jakobsen KS, et al. Ancient hybridizations among the ancestral genomes of bread wheat. Science. 2014;345(6194):1250092. 25035499 10.1126/science.1250092

[55] Wang X, Yan X, Hu Y, Qin L, Wang D, Jia J, et al. A recent burst of gene duplications in Triticeae. Plant Commun. 2022;3(2):12. 10.1016/j.xplc.2021.100268 PMC9073319 35529951

[56] Wang H, Xu F. Identification and expression analysis of the GLK gene family in tea plant (*Camellia sinensis*) and a functional study of CsGLK54 under low-temperature stress. Sci Rep. 2024;14(1):12465. 38816567 10.1038/s41598-024-63323-1 PMC11139860

[57] Li Z, Gao J, Wang B, Xu J, Fu X, Han H, et al. Rice carotenoid biofortification and yield improvement conferred by endosperm-specific overexpression of OsGLK1. Front Plant Sci. 2022;13:951605. 35909772 10.3389/fpls.2022.951605 PMC9335051

[58] Yeh S-Y, Lin H-H, Chang Y-M, Chang Y-L, Chang C-K, Huang Y-C, et al. Maize Golden2-like transcription factors boost rice chloroplast development, photosynthesis, and grain yield. Plant Physiol. 2022;188(1):442–59. 34747472 10.1093/plphys/kiab511 PMC9049120

[59] Zhang Q-w, Mao Y-y, Zhao Z-k, Hu X, Hu R, Neng-wen Y, Sun X, Sun F, Chen S, Jiang Y-x et al: A Golden2-like transcription factor, BnGLK1a, improves chloroplast development, photosynthesis, and seed weight in rapeseed. Journal of Integrative Agriculture 2024, 23(5):1481-1493.

[60] Du J, Zhao Z, Jin L, Huang L, Jin D, Zheng X, et al. Identification of a central regulator of ginkgolide biosynthesis in Ginkgo biloba that integrates jasmonate and light signaling. Proc Natl Acad Sci U S A. 2025;122(6):e240889112. 10.1073/pnas.2408891122 PMC11831150 39903108

[61] Sun T, Hazra A, Lui A, Zeng S, Wang X, Rao S, et al. GLks directly regulate carotenoid biosynthesis via interacting with GBFs in plants. New Phytol. 2025;246(2):645–65. 39953697 10.1111/nph.20457

[62] Yue H, Zhang H, Su N, Sun X, Zhao Q, Weining S, et al. Integrate small RNA and degradome sequencing to reveal drought memory response in wheat (*Triticum aestivum* L.). Int J Mol Sci. 2022;23(11):5917. 35682597 10.3390/ijms23115917 PMC9180835

[63] Rubio V, Linhares F, Solano R, Martín AC, Iglesias J, Leyva A, et al. A conserved MYB transcription factor involved in phosphate starvation signaling both in vascular plants and in unicellular algae. Genes Dev. 2001;15(16):2122–33. 11511543 10.1101/gad.204401 PMC312755

[64] Xiong B, Gong Y, Li Q, Li L, Mao H, Liao L, et al. Genome-wide analysis of the GLK gene family and the expression under different growth stages and dark stress in Sweet Orange (*Citrus sinensis*). Horticulturae. 2022;8(11):1076.

[65] Xie X, Lai W, Che X, Wang S, Ren Y, Hu W, et al. A SPX domain-containing phosphate transporter from *Rhizophagus irregularis* handles phosphate homeostasis at symbiotic interface of arbuscular mycorrhizas. New Phytol. 2022;234(2):650–71. 35037255 10.1111/nph.17973

[66] Liu B, Xu W, Niu Y, Li Q, Cao B, Qi J, et al. TaTCP6 is required for efficient and balanced utilization of nitrate and phosphorus in wheat. Nat Commun. 2025;16(1):1683. 39956820 10.1038/s41467-025-57008-0 PMC11830803

