# Supplementary material for: Genome-wide identification of the GLK gene family in wheat (Triticum aestivum L.) and analysis of expression responses in different environments
Source: BMC Genomics. 2026 Jan 20;27:78. doi: 10.1186/s12864-025-12456-2 (PMC12829200; doi:10.1186/s12864-025-12456-2)
Supplement: Supplementary file 1 — Supplementary Material 1. [file 12864_2025_12456_MOESM1_ESM.docx]

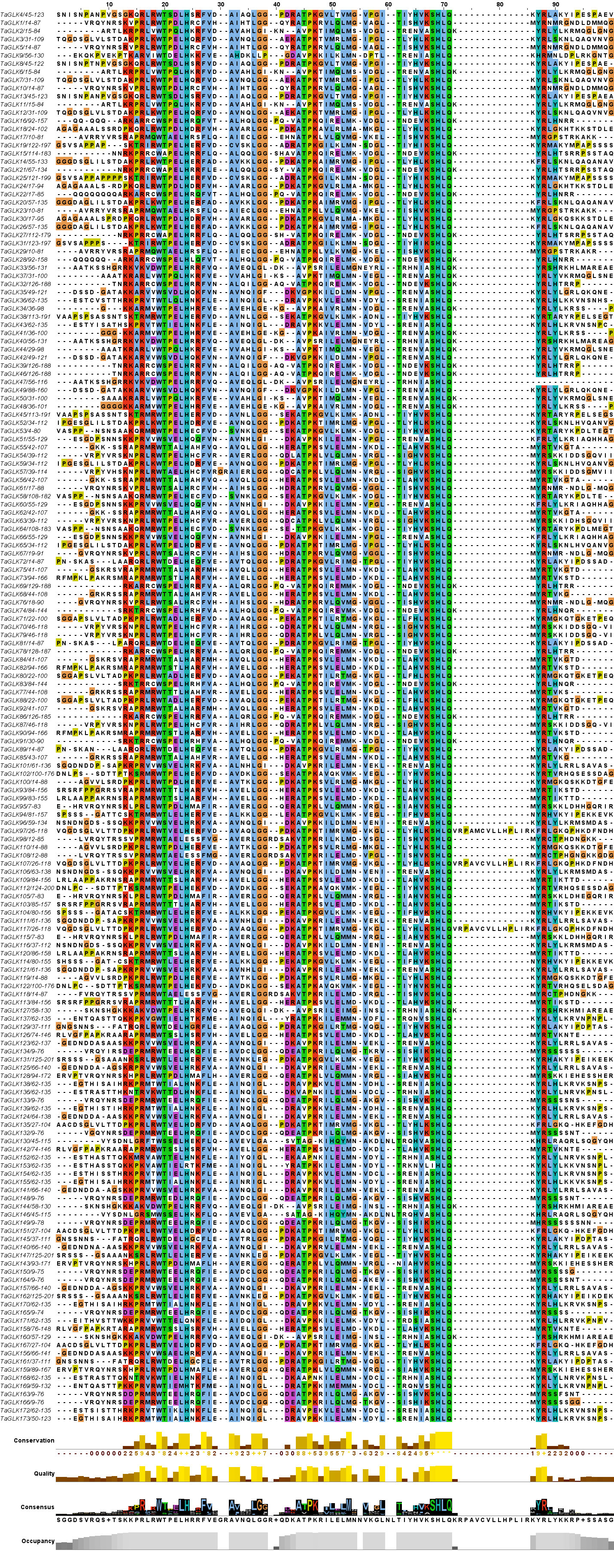


Fig. S1 Multiple sequence alignment of 173 TaGLK proteins


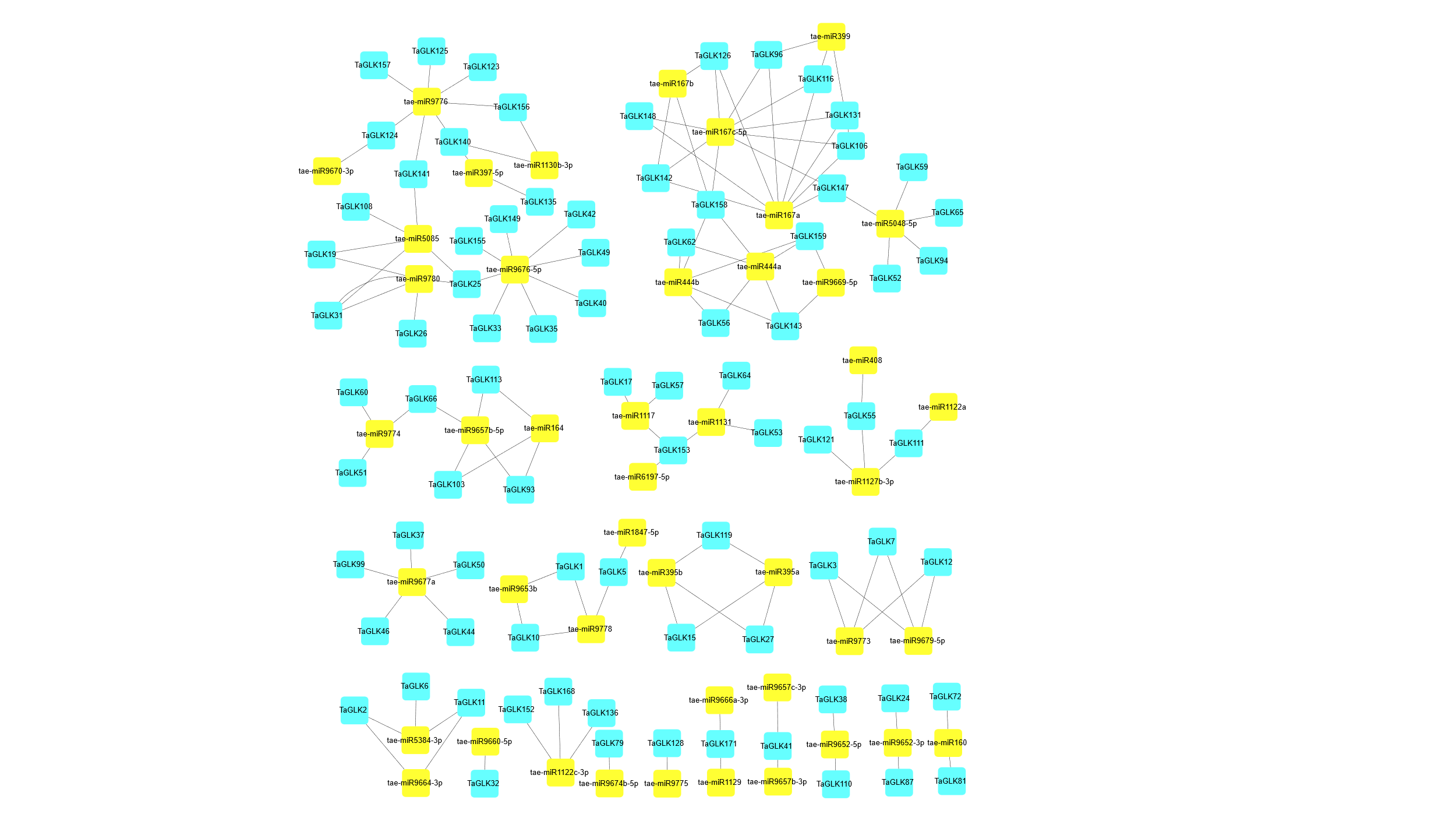


Fig. S2 Interaction network of target miRNAs and their *TaGLK* partners using Cytoscape.


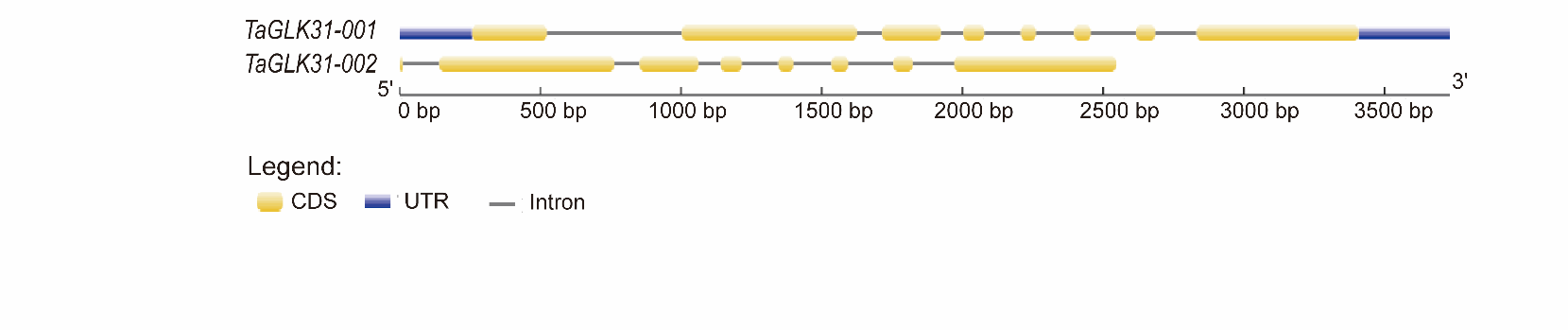


Fig. S3 The alternative splicing isoforms of the *TaGLK31* gene
